# Spontaneous Transvaginal Evisceration of the Small Bowel Two Years Post Laparoscopic Total Hysterectomy: A Case Report

**DOI:** 10.7759/cureus.86305

**Published:** 2025-06-18

**Authors:** Hanane Houmaid, Myriem Sali, Karam Harou, Hamid Asmouki, Abderraouf Soummani

**Affiliations:** 1 Obstetrics and Gynecology Department, Mohammed VI University Hospital Center, Cadi Ayyad University, Marrakesh, MAR

**Keywords:** bowel evisceration, laparoscopic hysterectomy complications, surgical emergency, total hysterectomy, vaginal cuff dehiscence

## Abstract

Vaginal evisceration after hysterectomy, although rare, is a serious postoperative complication. It requires rapid diagnosis and management. The small bowel is the most common organ of evisceration, but prolapse of the omentum, appendix, and fallopian tubes has also been described. We report a case of a 51-year-old postmenopausal patient who presented to the gynecologic emergency department with vaginal evisceration after dancing at a family party. She felt an acute abdomino-pelvic pain with vaginal lump sensation. Her medical history included a laparoscopic hysterectomy for benign pathology two years before. The gynecological examination revealed vaginal evisceration of intestinal loops by a rupture of the cuff of the vagina. A laparotomic dehiscence repair was performed, with preservation of the entire intestine, which was undamaged, and the patient was discharged three days after.

## Introduction

Vaginal small bowel evisceration is a rare complication after total hysterectomy. It represents a serious gynecological emergency, and any negligence can threaten the woman's life. It can be complicated by bowel ischemia, ileus, and peritonitis. It was first reported in 1864 by Hypernaux et al. [1], and then by McGregor in 1907 [2]. It is very rare, and to date, fewer than 150 cases have been described worldwide [3-14].

The exact etiology remains unknown but several risk factors have been reported, such as advanced woman age, postmenopausal tissue atrophy, history of vaginal or abdominal surgery, suboptimal surgical technique, history of prior radiation therapy, sexual assault, and enterocele [15]. It may be spontaneous or preceded by an increase in abdominal pressure or a vaginal trauma that weakens the scarred vaginal dome, where there is a decreased in vascularization and vaginal atrophy.

We present a case of transvaginal evisceration of the small bowel in a patient with a past surgical history two years ago of a laparoscopic assisted vaginal hysterectomy for a benign pathology. This case highlights a rare and serious postoperative complication of total hysterectomy, small bowel evisceration through vaginal cuff dehiscence. Clinicians must be aware of the need to perform a prompt diagnosis and to coordinate with multiple specialists for good management to reduce its morbidity and mortality.

## Case presentation

A post-menopausal 51-year-old female patient (gravidity: 4, parity: 4, 4 uneventful vaginal live births) presented to the obstetrics and gynecology emergency department with acute abdominal-pelvic pain and a sensation of organ prolapse through her vagina, without nausea, vomiting or fever. She reported feeling the pain one hour before her admission and immediately after dancing at a family party. The patient’s past surgical history included a laparoscopic assisted vaginal hysterectomy two years ago for a benign pathology (myomatous uterus). The intervention was uneventful, and the postoperative follow-up was good.

**Figure 1:**
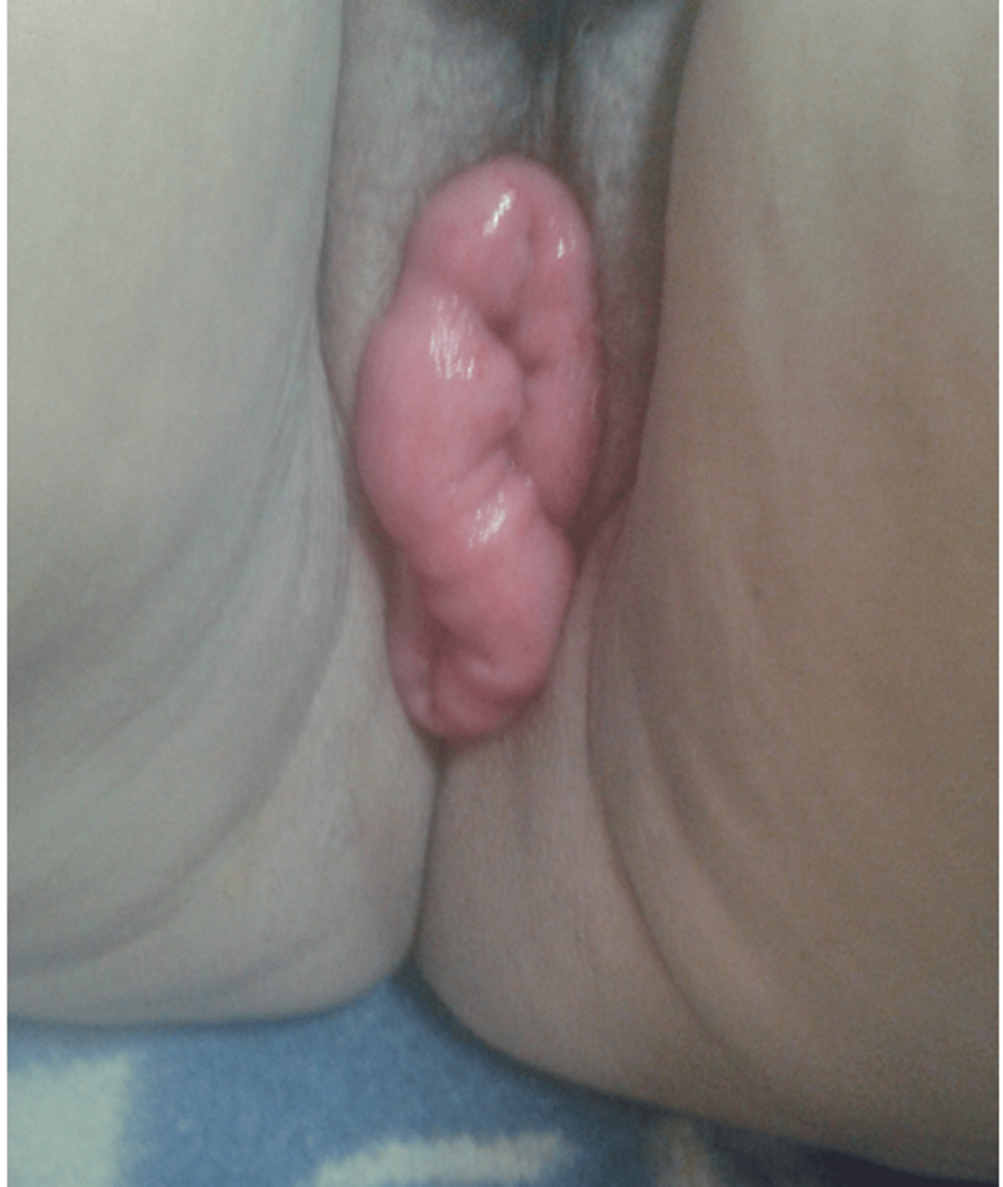
Clinical image of transvaginal evisceration of the small bowel

On examination, vaginal hernia of the small intestine was noted; it affected the ileum for a length of approximately 20 cm; without any changes in color, peristalsis, or temperature (Figure 1).

There was no bleeding or vaginal discharge. The patient was conscious, her vital signs and blood investigations were normal with blood pressure of 135/87 mmHg, pulse rate of 91 beats per minute, respiratory rate of 18 breaths per minute, and temperature of 37°C. Laboratory investigations done included full blood count, which showed normal WBC of 5 10^9^/L, hemoglobin of 12g/dl, and platelets of 139 10^9^/l, C-reactive protein less than 0.6 mg/dl. She received antibiotic prophylaxis, 2 g of amoxicillin/clavulanic acid.

The bowel was covered with warm, sterile, saline-soaked pads. After consulting the anesthesia and surgeon teams, and given the absence of a laparoscopy column, an open midline laparotomy approach was urgently decided to prevent complications; it was carried out jointly between the gynecologic and the surgeon teams under general anesthesia.

The perioperative abdomen-pelvic exploration was unremarkable; there were no adhesions, and fortunately, the bowel remained viable and was reintroduced to the abdomen. After abundant washing with sterile saline, the vaginal cuff was carefully excised to remove all necrotic tissue and full-thickness separated sutures were performed using delayed absorbable monofilament suture, 0-Polydioxanone (Ethicon Endo-Surgery, Cincinnati, Ohio, United States). The postoperative follow-up was uneventful, and the patient was discharged three days later. The patient experienced an uncomplicated postoperative recovery.

On postoperative day 20, a speculum examination demonstrated an intact suture line at the vaginal vault. Follow-up assessment at three, six, and 12 months postoperatively demonstrated complete healing, with no evidence of dehiscence or prolapse.

## Discussion

Hysterectomy is the removal of the uterus; it’s the most common surgical intervention in gynecology. Hysterectomy can be performed for benign or malignant pathology. Abdominal, vaginal, or laparoscopic hysterectomy or a combined approach, depending on multiple factors such as the patient’s age, uterine size and mobility, body mass index, nulliparity, history of abdominal or pelvic surgery, surgeon’s skills and the woman’s choice [16,17]. It’s a surgical procedure that can cause many complications; DeNeradis et al. classified them as perioperative to postoperative [18]. Vaginal cuff dehiscence and evisceration of the small bowel is a postoperative complication special to total hysterectomy; it usually happens several weeks to several months after the hysterectomy, or even several years [9].

The reported incidence of transvaginal evisceration after hysterectomy has been reported at 0.28%, though the true rate is likely underestimated [19]. The small bowel represents the most common intra-abdominal organ to eviscerate [9,20,21]. Although it is a rare complication, it can lead to heavy health consequences with serious morbidity and mortality. In the present case, we hypothize that spontaneous small bowel evisceration was caused by a gradual weakening of the vaginal vault, postmenopausal tissue atrophy and a sudden increase in intra-abdominal pressure.

It seems that closing the vaginal cuff is an important step during hysterectomy. It separates the aseptic peritoneal cavity and the septic vagina, so any dehiscence at its level will put these two compartments in direct communication. A delay in diagnosis and management may lead to bowel injury, necrosis, sepsis, and sometimes death (mortality up to 10%) [22,23]. There is a different surgical approach to managing this complication; the choice of an approach depends on the patient’s hemodynamic stability, bowel viability, surgeon’s skills, and available tools (laparoscopic column) [24,25]. Laparotomic, vaginal, or laparoscopic approaches can be used in order to repair vaginal cuff dehiscence, and to fix complications like ischemia, perforation, or bowel necrosis. In our case, we chose the first approach, due to the unavailability of a laparoscopic column and because it offers good surgical exposure of the exteriorized bowel and the rest of the intestine, and makes it easier to reintroduce it in the peritoneal cavity, and to excise the ischemic part if there is any.

Surgical management should be as prompt as possible to avoid intestinal ischemia. When there is no sign of peritonitis, ischemia, or necrosis, and full bowel reduction is possible, a vaginal approach may be considered. Nonetheless, it does not allow for a complete evaluation of the entire bowel and abdominal cavity. Laparotomy offers a more thorough assessment of the abdomen-pelvic structures before vault repair but exposes the patient to higher morbidity compared with a combined laparoscopic-vaginal approach [9].

Rupture of the vaginal vault after total hysterectomy is a rare and life-threatening complication, compromising the intra-abdominal viscera. It requires urgent intervention to avoid local complications such as ischemic bowel and systemic complications such as generalized peritonitis. Physicians should remain vigilant of any acute pelvic pain, vaginal bleeding, or discharge in a woman who has undergone a hysterectomy in her far or near medical history. The treatment of the vaginal cuff dehiscence is always surgical, although several approaches have been described. Sexual activity becomes a challenge for these patients, and to prevent traumas and especially recurrences, psychosexual therapy is strongly recommended [26].

## Conclusions

Transvaginal small bowel evisceration is a surgical emergency requiring urgent multidisciplinary intervention to prevent complications. Prompt evaluation of bowel viability is essential in guiding management. If the bowel appears viable, vaginal reduction and repair may suffice. However, a combined abdominal (open or laparoscopic) and vaginal approach is often preferred to allow thorough bowel inspection and accurate assessment of the vaginal vault defect, ensuring safe and effective treatment. This case has been reported to highlight a rare and serious postoperative complication of total hysterectomy. Small bowel evisceration through vaginal cuff dehiscence is a surgical emergency, and a potentially fatal condition. Clinicians must be aware of this to perform a prompt diagnosis and to coordinate with multiple specialists for good management to reduce its related morbidity and mortality.
